# A Novel Allogeneic Rituximab-Conjugated Gamma Delta T Cell Therapy for the Treatment of Relapsed/Refractory B-Cell Lymphoma

**DOI:** 10.3390/cancers15194844

**Published:** 2023-10-04

**Authors:** Hao-Kang Li, Tai-Sheng Wu, Yi-Chiu Kuo, Ching-Wen Hsiao, Hsiu-Ping Yang, Chia-Yun Lee, Pei-Ju Leng, Yun-Jung Chiang, Zih-Fei Cheng, Sen-Han Yang, Yan-Liang Lin, Li-Yu Chen, Ciao-Syuan Chen, Yu-Ju Chen, Shih-Chia Hsiao, Sai-Wen Tang

**Affiliations:** 1 Acepodia Biotech Inc., Alameda, CA 94502, USA; howard@acepodiabio.com (H.-K.L.); ericwu@acepodiabio.com (T.-S.W.); yichiu@acepodiabio.com (Y.-C.K.); stephanie@acepodiabio.com (C.-W.H.); cindy@acepodiabio.com (H.-P.Y.); sylvia@acepodiabio.com (C.-Y.L.); winni.leng@acepodiabio.com (P.-J.L.); morris.chiang@acepodiabio.com (Y.-J.C.); zihfei@acepodiabio.com (Z.-F.C.); samshyang@acepodiabio.com (S.-H.Y.); yanliang@acepodiabio.com (Y.-L.L.); 2 Institute of Chemistry, Academia Sinica, Taipei 11529, Taiwan; clu0506@gmail.com (L.-Y.C.); c27221@gmail.com (C.-S.C.); yujuchen@gate.sinica.edu.tw (Y.-J.C.); 3 Department of Chemistry, National Taiwan University, Taipei 10617, Taiwan

**Keywords:** gamma delta T cells, γδ T, antibody–cell conjugation, ACE1831, cancer cell therapy, rituximab, CD20, bio-orthogonal chemistry

## Abstract

**Simple Summary:**

CD20-targeting CAR T cells have shown remarkable clinical outcomes in patients with B-cell malignancies. However, the manufacturing processes usually involved in autologous cell collection and gene editing limit their usage and development across the board. Here, we developed an allogenic cell product ACE1831, Vγ9Vδ2 T (γδ2 T) cells equipped with anti-CD20 antibody, using antibody–cell conjugation (ACC) technology. ACE1831 elicits a specific and potent anti-tumor activity against CD20-expressing cancer cells in vitro and in vivo with no abnormal clinical observation. We further found that the ACC-linked antibody/receptor complex stimulated T cell activation upon recognizing the antigen on cancer cells. To summarize, our data show the promise of a novel combination of an ACC platform with γδ2 T cells in allogenic immunotherapy against relapsed/refractory B-cell lymphoma.

**Abstract:**

Chimeric antigen receptor T cell (CAR-T) therapy has been applied in the treatment of B-cell lymphoma; however, CAR-T manufacturing requires virus- or non-virus-based genetic modification, which causes high manufacturing costs and potential safety concerns. Antibody–cell conjugation (ACC) technology, which originated from bio-orthogonal click chemistry, provides an efficient approach for arming immune cells with cancer-targeting antibodies without genetic modification. Here, we applied ACC technology in Vγ9Vδ2 T (γδ2 T) cells to generate a novel off-the-shelf CD20-targeting cell therapy ACE1831 (rituximab-conjugated γδ2 T cells) against relapsed/refractory B-cell lymphoma. ACE1831 exhibited superior cytotoxicity against B-cell lymphoma cells and rituximab-resistant cells compared to γδ2 T cells without rituximab conjugation. The in vivo xenograft study demonstrated that ACE1831 treatment strongly suppressed the aggressive proliferation of B-cell lymphoma and prolonged the survival of tumor-bearing mice with no observed toxicity. Mass spectrometry analysis indicated that cell activation receptors including the TCR complex, integrins and cytokine receptors were conjugated with rituximab. Intriguingly, the antigen recognition of the ACC-linked antibody/receptor complex stimulated NFAT activation and contributed to ACE1831-mediated cytotoxicity against CD20-expressing cancer cells. This study elucidates the role of the ACC-linked antibody/receptor complex in cytotoxicity and supports the potential of ACE1831 as an off-the-shelf γδ2 cell therapy against relapsed/refractory B-cell lymphoma.

## 1. Introduction

The success of chimeric antigen receptor T cells (CAR-T) for the treatment of acute lymphoblastic leukemia and non-Hodgkin’s lymphoma is a milestone in cell therapy; however, CAR-T therapy has been associated with severe adverse events including fatal cytokine release syndrome and graft-versus-host disease due to the primary population of αβ T cells in the end product [1,2]. γδ T cells represent a unique T cell subpopulation containing a T cell receptor (TCR) composed of γ and δ chains with diverse structures and functional heterogeneity, which are enriched in many peripheral tissues including peripheral blood and contribute to spatial immune responsiveness in an innate- and adaptive-like manner [3,4,5,6]. Compared to αβ T cells, γδ T cells, derived from donor-derived peripheral blood, recognize their target cells in an MHC/HLA-independent manner, leading to a low or absent risk for alloreactivity and graft-versus-host disease (GvHD), thus enabling their allogeneic clinical application for malignancies without causing severe side effects [7,8,9]. Vγ9Vδ2 T cells (γδ2 T cells), the major subtype of γδ T cells, constitute 1 to 5% of circulating lymphocytes in healthy adults and are characterized as a hybrid of the αβ T and NK cell gene signature [5,10]. The infusion of expanded allogenic γδ2 T cells predicts good clinical outcomes in patients with solid and liquid tumors [10,11]. Recently, applications of CAR-modified γδ T cells further improved their efficacy in the eradication of diverse malignancies including human GD2-expressing neuroblastoma and CD19-expressing Burkitt’s lymphoma [12,13,14].

The innovation of click chemistry and bio-orthogonal chemistry, which provides the chemical reactions to link molecules under ambient or biological conditions, led to a Nobel Prize being awarded in 2022. For biomedical research, bio-orthogonal chemistry enables the selective modification of biomolecules and living cells without interfering in biological processes [15]. An effective cell surface modification with single-strand DNA through the bio-orthogonal Staudinger reaction has been reported, which requires multiple days of exposure to achieve a sufficient modification [16,17]. Hsiao et al. performed a direct and rapid bio-conjugation to modify cell surface proteins with (N-hydroxysuccinimide) NHS-DNA conjugates within a few hours and applied DNA hybridization to direct living cells to surface coated with complementary DNA [18]. Based on the optimized bio-orthogonal reaction for cell surface modification, antibody–cell conjugation (ACC) technology was developed and applied to link cancer-targeting antibodies on immune cells such as cytokine-induced killer and NK cells [19,20]. The successful application of ACC technology in cancer treatment was demonstrated by the specific cancer antigen recognition and superior anti-cancer potency of antibody-armed immune cells [20], leading to a clinical trial for HER2-expressing tumors.

To extend ACC application to T cell-based cancer therapy, here, we examine the compatibility of ACC technology with PBMC-derived γδ2 T cells. In this study, for the first time, ACC technology is applied to γδ2 T cells to generate rituximab-conjugated γδ2 T cells, ACE1831, and their potency against CD20-expressing cancer cells is evaluated. Rituximab-linked surface proteins of γδ2 T cells are identified through mass spectrometry analysis. Our investigation of T cell activation mediated by the antibody-antigen recognition of rituximab-linked receptors elucidates the potential of the ACC-mediated activation mechanism of ACE1831 for the first time. This study provides evidence to support ACE1831’s role as a potent, off-the-shelf cell therapy against CD20-expressing cancers.

## 2. Materials and Methods

### 2.1. Antibodies, Cell Lines, and Mice

The antibodies used in this study were obtained from Biolegend (San Diego, CA, USA), except for the anti-F(ab’)2 antibody, which was from Jackson ImmunoResearch Labs (West Grove, PA, USA). Daudi, Raji, and K562 were obtained from ATCC (Manassas, VA, USA) and cultured accordingly. Rituximab-resistant Raji cells were generated and maintained in the presence of 80 μg/mL of rituximab as previously described [21], and the resistance of the rituximab-resistant Raji cells to rituximab was examined (Supplementary Figure S1). Female SCID–Beige mice (CB17.Cg-PrkdcscidLystbg-J/Crl, 6–10 weeks old) were purchased from Charles River Laboratories (Wilmington, MA, USA) and housed according to the regulations of the Institutional Animal Care and Use Committee (IACUC) of the contract research organizations.

### 2.2. ACE1831 Generation

Peripheral blood mononuclear cells (PBMCs) were collected from healthy donors and used for ACE1831 generation. γδ2 T cells were specifically activated, selected and expanded through the proprietary processes, as described previously (patent WO2022221506). The conjugation of anti-CD20 antibody rituximab with γδ2 T cells was performed based on the protocol previously described [18,19]. In brief, NHS-DNA conjugates, generated from a 5′-thiolated single-strand DNA linker-1 and linker-2 with complementary sequences, were conjugated with γδ2 T cells and rituximab, respectively. After washing with DPBS, linker-1-conjugated γδ2 T cells and linker-2-conjugated rituximab were mixed at room temperature to allow for complementary DNA hybridization. After washing out the free linker-2-conjugated rituximab, the rituximab-linked γδ2 T cells were cryopreserved and stored in liquid nitrogen for further analysis.

### 2.3. Flow Cytometry Analysis

Cell suspensions derived from cells stained with fluorescent-dye-conjugated antibodies after 2 washes with DPBS were analyzed with an Attune NxT flow cytometer with the Attune NxT software 3.1.0 installed. Propidium iodide staining was used to determine the viability of the cells. γδ2 T cells were defined by gating CD3+ and TCRγδ2+ populations, and the efficiency of rituximab conjugation for the γδ2 T cells was examined through staining with an R-phycoerythrin-coupled anti-F(ab’)2 antibody (Jackson ImmunoResearch Labs, West Grove, PA, USA).

To examine their CD20 binding capacity, the cells were incubated with 0.001, 0.01, 0.1, 1, 10 and 100 μg/mL of recombinant human CD20-His protein (ACROBiosystems, Newark, DE). After washing with DPBS, the cells were stained with an FITC-conjugated anti-6X His tag antibody (Abcam, Waltham, MA, USA). The stained cells were washed with DPBS, and the CD20-bound cell population was identified through flow cytometry analysis. 

The cells were prepared and analyzed for the effector function markers CD107a, granzyme B and IFNγ as previously described [20]. The fold change in the mean fluorescence intensity (MFI) of CD107a, granzyme B and IFNγ in the effector cells was calculated relative to γδ2 T cells without coculturing with target cancer cells (set as 1).

### 2.4. In Vitro Cytotoxicity

The CellTiter-Glo 2.0 Cell Viability Assay (Promega, Madison, WI, USA) was applied according to the manufacturer’s instructions. In brief, CD20-positive Daudi, Raji and rituximab-resistant Raji and CD20 negative K562 cancer cell lines, as well as donor PBMCs, were seeded in white opaque plates and incubated with the effector cells at the indicated effector € to target (T) ratios at 37 °C for 4 h. For the TCRγδ blocking experiments, the effector cells were preincubated with 1 μg/mL of TCRγδ blocking antibody (clone 5A6.E9, Invitrogen, Waltham, MA, USA) for 1 h before co-incubation with Raji cells. The cultures were then mixed with the reaction substrate, and the luminescence was detected using a Synergy H1 Hybrid Multi-Mode Reader (Agilent, Santa Clara, CA, USA). The absorbance of the coculture of effect€(E) and target (T) cells, the effector alone, and the target alone was designated as ET, E and T. The % of cytotoxicity of each group was calculated using the equation [1 − (ET − E)/T] × 100.

### 2.5. Animal Studies

For the evaluation of in vivo efficacy, 1 × 10^5^ firefly luciferase-expressing Raji cells (raji-FLuc, Creative Biogene, New York, NY, USA) were intravenously implanted into female SCID–Beige mice on Day 0. ACE1831 (1 × 10^7^), γδ2 T cells (1 × 10^7^) and a serum-free medium (Vehicle) Vehicle were administered intravenously through the tail vein twice per week for two weeks. The tumor luminescence signals were captured using an Ami HTX spectral instrument (Accela, San Ramon, CA, USA) and analyzed with Aura software (Version 4.0.7). The survival status of the mice was recorded and analyzed using the Kaplan–Meier survival estimation method. All mice were sacrificed on Day 95.

### 2.6. Computational Interpretation of the Cell Surface Proteome

The rituximab-conjugated surface proteins of γδ2 T cells were immunoprecipitated and analyzed with mass spectrometry, as previously described [18], using Proteome Discoverer software v2.1 (Thermo Fisher, Hayward, CA, USA). With Proteome Discoverer, we searched the resulting spectra against the UniProt human database (accessed March 2021) with common contaminant protein sequences included and all the corresponding reversed sequences using the SEQUEST algorithm. The precursor mass tolerance was set to ±10 ppm, and the fragment mass tolerance was set to ±0.02 Da. The carbamidomethylation of cysteine (+57.021 Da) was set as a static modification. The differential modifications were set to a maximum of three, and the following were used: for the oxidation of methionine, +15.995 Da; for the phosphorylation of serine, tyrosine and threonine, +79.9663; and for the heavy lysine and arginine, +6.020 Da. Enzyme specificity was set to trypsin, allowing for up to two missed cleavages. The minimum required peptide length was set to seven amino acids, and the data were filtered to a 1% peptide and protein false discovery rate using Percolator.

Peak area quantification was performed using the Proteome Discoverer label-free module. Protein candidates with an ACE1831/un-conjugated γδ2 T cell ratio larger than 550 for the batches and an average frequency less than 0.3 were included. The frequency of the AP-MS contaminants was extracted determined the CRAPome database. The protein localization count was determined using the UniProtKB/Swiss Prot protein knowledge base. Within the UniProtKB/Swiss-Prot protein localization groupings, the extracellular exosome, cell surface, plasma membrane, extracellular space and membrane were categorized as “membrane structure” categories. For the other localization groupings, no further categorization was performed. The protein candidates’ cellular component, biological process, KEGG pathway GO term and p-value were analyzed with the Gene Ontology (GO) Consortium and the Database for Annotation, Visualization, and Integrated Discovery (DAVID, v6.8).

### 2.7. Jurkat-NFAT-Luc Reporter Assay

Jurkat-NFAT-Luc cells (Invivogen, San Diego, CA, USA) were conjugated with different amounts of linker-1, and linker-2 rituximab was further bound to linker-1-conjugated Jurkat-NFAT-Luc cells. The rituximab-linked Jurkat NFAT Luc cells were incubated with Raji cells at an E:T ratio of 5 and 10 at 37 °C for 16 h, followed by the addition of 100 µL luciferase substrate (Invivogen) to each well. The luminescence signal was immediately recorded with a Biotek Synergy HTX multimode reader.

### 2.8. Statistical Analysis

The data from the cytotoxicity assay and flow cytometry were analyzed with an unpaired Student’s *t* test. The tumor luminescence signals between each treatment in the animal studies were analyzed with a two-way ANOVA.

## 3. Results

### 3.1. Rituximab Conjugation on γδ2 T Cells Using ACC Technology

**Figure 1:**
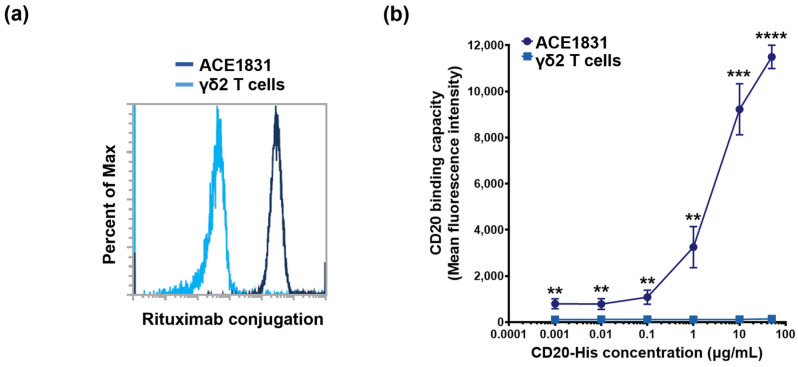
Rituximab conjugation confers γδ2 T cells with CD20 binding capacity. (**a**) A representative histogram of rituximab conjugation is illustrated. DNA linker-1 and linker-2 were conjugated with γδ2 T cells and rituximab, respectively. Linker-1-conjugated γδ2 T cells and linker-2-conjugated rituximab were mixed and ACE1831, rituximab-linked γδ2 T cells, were generated through DNA hybridization. Un-conjugated γδ2 T cells and ACE1831 were stained with R-phycoerythrin-coupled anti-F(ab’)2 antibody to determine the rituximab conjugation efficiency through flow cytometry. Un-conjugated γδ2 T cells (light blue line) represent negative staining, and efficient rituximab conjugation on ACE1831 (dark blue line) is shown. Percent of Max is the highest point of each peak of the overlaid histogram derived from ACE1831 and γδ2 T cells. (**b**) CD20 binding capacity of ACE1831 and γδ2 T cells was determined through flow cytometry analysis. The cells were incubated with 0.001, 0.01, 0.1, 1, 10 and 100 μg/mL of human CD20-His recombinant protein, and the CD20-bound cell population was identified through staining with Fluorescein-coupled anti-6X His tag antibody. The study was performed in triplicate in five different experiments, and the representative results are shown. Statistical analysis was performed using the *t* test. **, *p* < 0.01; ***, *p* < 0.001; ****, *p* < 0.0001.

To equip the γδ2 T cells with CD20-targeting specificity, ACC technology was applied to conjugate the γδ2 T cells, expanded from the PBMCs of healthy donors, with the CD20-targeting antibody rituximab. In the first reaction, the γδ2 T cells and rituximab were conjugated with DNA linker-1 and linker-2 with complementary sequences, respectively. After the DNA hybridization reaction, the linker-1-conjugated γδ2 T cells and linker-2-conjugated rituximab were connected to form rituximab-linked γδ2 T cells, defined as ACE1831. Rituximab conjugation could be detected on approximately 100% of the ACE1831, revealing that ACC technology successfully and efficiently links antibodies to γδ2 T cells (Figure 1a). To examine the specificity of ACE1831 binding to CD20, the binding capacity of ACE1831 to CD20-His recombinant protein at designated concentrations was analyzed through flow cytometry. As shown in Figure 1b, the CD20 protein bound on the surface of ACE1831 was significantly increased in a dose-dependent manner compared to the un-conjugated γδ2 T cells.

### 3.2. Enhanced Cytotoxicity of ACE1831 against CD20-Expressing Cancer Cells

**Figure 2:**
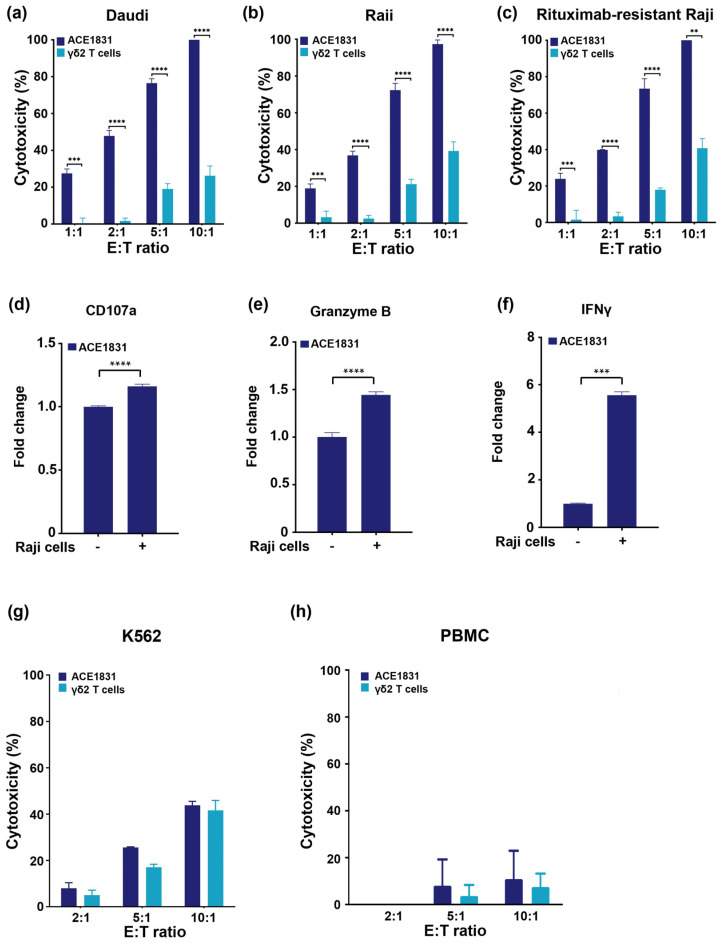
Rituximab conjugation confers γδ2 T cells with superior cytotoxicity against CD20-expressing cancer cells. (**a**–**c**) ACE1831 and γδ2 T cells were co-incubated with CD20-expressing cancer cells. (**a**) Daudi, (**b**) Raji and (**c**) rituximab-resistant Raji cells at effector to target (E:T) ratios of 1:1, 2:1, 5:1 and 10:1, analyzed via CellTiter-Glo^®^ luminescent cell viability assay after 4 h of co-incubation. (**d**) CD107a, (**e**) granzyme B and (**f**) IFNγ of ACE1831 in the absence and presence of Raji cells at an E:T ratio of 2:1 after 2 h of incubation were analyzed via flow cytometry. Each group was performed in triplicate from two donor lots, and the representative results are shown. Statistical analysis was performed using the *t* test. **, *p* < 0.01; ***, *p* < 0.001; ****, *p* < 0.0001. (**g**,**h**) ACE1831 and γδ2 T cells were co-incubated with (**g**) CD20-negative K562 and (**h**) donor PBMC cells at effector to target (E:T) ratios of 2:1, 5:1 and 10:1 and analyzed using a CellTiter-Glo^®^ luminescent cell viability assay after 4 h of co-incubation.

To examine the potency of ACE1831 against CD20-expressing cancer cells, ACE1831 was co-incubated with CD20 expressing the Burkitt’s lymphoma cell lines Daudi and Raji, and the cytotoxicity was measured using a CellTiter-Glo^®^ luminescent cell viability assay. As shown in Figure 2a,b, ACE1831 exhibited significantly enhanced cytotoxicity against Daudi and Raji cells compared to the un-conjugated γδ2 T cells at effector (E) to target (T) ratios of 1:1, 2:1, 5:1 and 10:1. Of note, ACE1831 showed superior cytotoxicity against rituximab-resistant Raji cells in a similar magnitude to Raji cells (Figure 2c), indicating that ACE1831 elicits efficient anti-cancer potency to B-cell lymphoma cells independent of rituximab resistance. CD107a, granzyme B and IFNγ have been reported as effector function markers for the cytolytic activity of γδ T cells and other cytotoxic immune cells when encountering cancer cells [22,23,24]. Our flow cytometry analysis showed a significant increase in the CD107a and granzyme B levels of ACE1831 when encountering Raji cells (Figure 2d,e). Furthermore, the IFNγ level of ACE1831 was significantly enhanced through its coculturing with Raji cells (Figure 2f), while IL-6 secretion remained undetectable in both the ACE1831 and γδ2 T cells. These results suggest that ACE1831 is activated when targeting CD20-expressing cancer cells.

The specificity of ACE1831 cytotoxicity was examined through the co-incubation of ACE1831 or un-conjugated γδ2 T cells with CD20-negative lymphoblast K562 cells. No significant difference in cytotoxicity against K562 between the ACE1831 and un-conjugated γδ2 T cells at various E:T ratios was observed (Figure 2g), indicating that ACE1831 has specific cytotoxicity to CD20-expressing cells and a similar basal cytotoxicity to CD20-negative cancer cells, as compared to un-conjugated γδ2 T cells. It was noted that both the ACE1831 and γδ2 T cells did not exhibit marked cytotoxicity to PBMCs from healthy donors (Figure 2h). Additionally, the induction of cytokine release and/or adverse effects of ACE1831 treatment were assessed through a mixed lymphocyte reaction assay. The coculture of ACE1831 with PBMCs from healthy donors showed no significant immunogenic potential in the unstimulated and stimulated conditions (Figure S2). These results suggest that rituximab conjugation on γδ2 T cells using ACC technology provides specific potency against CD20-expressing cancer cells without a loss of basal anti-cancer capacity.

### 3.3. In Vivo Potency of ACE1831 against CD20-Expressing Cancer Cells

**Figure 3:**
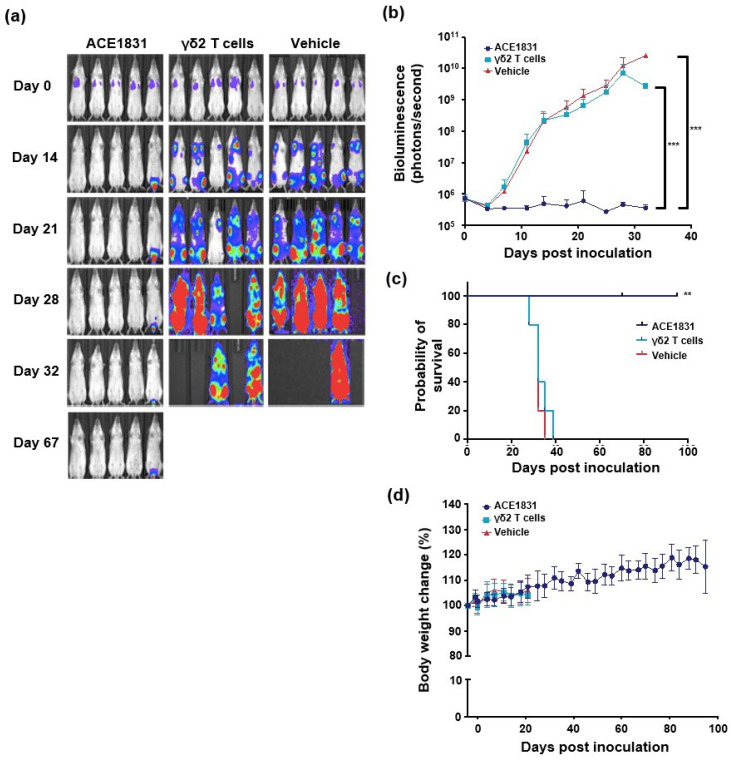
ACE1831 shows superior potency against CD20-expressing cancer cells in vivo. Four doses of intravenously delivered ACE1831 effectively suppressed tumor growth. Tumor-bearing SCID–Beige mice were treated with ACE1831, γδ2 T cells and a Vehicle (serum-free medium) twice per week for two weeks. (**a**) Tumor burden of mice (n = 5 per group) was determined through bioluminescence imaging. (**b**) The bioluminescence intensity of the tumor burden is presented as mean values ± SD. The difference in mean tumor burden between groups was examined using a two-way ANOVA. ***, *p* < 0.001. (**c**) The survival rate of mice with different treatments was analyzed using the Kaplan–Meier method. **, *p* < 0.01. (**d**) The body weight of each group of mice is presented as mean value ± SD.

To examine the cytotoxicity of ACE1831 against CD20-expressing cancer cells in vivo, ACE1831 and γδ2 T cells were infused twice weekly for two weeks into Raji-FLuc-bearing SCID–Beige mice, and the bioluminescence signal, body weight, clinical signs and survival status were monitored. The bioluminescence results revealed that four doses of ACE1831 treatment eliminated Raji cells from the mice and controlled Raji cell growth, while the un-conjugated γδ2 T cell- or Vehicle-treated groups showed an aggressive growth of Raji cells (Figure 3a,b). The ACE1831-treated mice survived throughout the study, whereas the γδ2 T cells or Vehicle-treated mice died before 40 days post-Raji cell inoculation (Figure 3c). The strong potency of one dose of ACE1831 treatment against Raji cells could be observed, and 40% of the ACE1831-treated mice survived over 120 days (Figure S3). Both studies showed that ACE1831 treatment significantly prolonged the survival of the mice compared to the Vehicle- and γδ2 T cell-treated groups (Figure 3c and Figure S3c). No abnormalities in body weight or clinical signs in the ACE1831-treated group were observed, and the clinicopathological examination revealed no ACE1831-related toxicology in the acute and recovered phases (Figure 3d and Figure S3). These results demonstrate the superior in vivo potency of ACE1831 against CD20-expressing cancer cells.

### 3.4. Identification of Antibody-Linked Surface Proteins of ACE1831 

**Figure 4:**
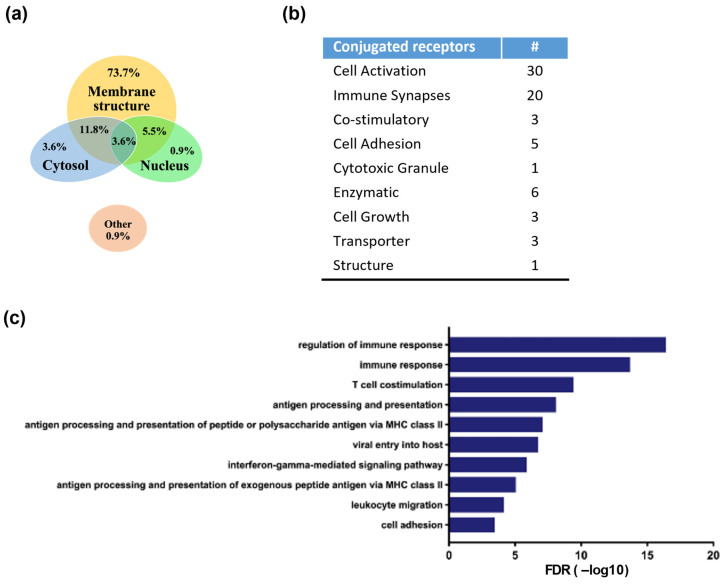
Identification and functional analysis of rituximab-conjugated proteins using mass spectrometry. Rituximab-linked proteins of ACE1831 were immunoprecipitated and analyzed using mass spectrometry. Protein candidates with ACE1831/un-conjugated γδ2 T cell ratios larger than 550 and an average frequency less than 0.3 were identified as rituximab-linked proteins. The cellular component, biological process, KEGG pathway GO term and p-value were analyzed with the GO Consortium and the Database for Annotation, Visualization, and Integrated Discovery. (**a**) Proportion of identified rituximab-linked proteins in the cellular localization. (**b**) Functional categorization of rituximab-linked proteins. “#” stands for the number of conjugated receptors in each functional category. (**c**) Top 10 categories of biological processes among the identified rituximab-linked proteins.

To identify the antibody-linked proteins, ACE1831 and un-conjugated γδ2 T cells were lysed, and the rituximab-linked proteins were enriched through co-immunoprecipitation with protein A beads and DNase digestion, followed by mass spectrometry analysis, as previously described [19]. Seventy-two proteins with ACE1831/γδ2 T cell signals larger than 550 and an average frequency less than 0.3 were identified as rituximab-linked proteins. The cellular component, biological process and KEGG pathway GO term of the identified rituximab-linked proteins were analyzed with the GO Consortium and the Database for Annotation, Visualization, and Integrated Discovery. Most rituximab-linked proteins were localized at the membrane structure (73.7%), and few were in the cytoplasm (3.6%) and nucleus (0.9%) (Figure 4a). The protein functional analysis indicated that the main components of the identified surface proteins were related to cell activation (*n* = 30) and the immune synapse (*n* = 20), including the TCR complex (TCRδ, CD3δ and CD3γ), integrins and cytokine receptors (Figure 4b), and the top 10 categories of biological process Gene Ontology (GO) terms were mainly involved in the regulation of immune responses (Figure 4c).

### 3.5. T Cell Activation and Cytotoxicity Mediated by Antigen Recognition of ACC-Linked Antibody 

**Figure 5:**
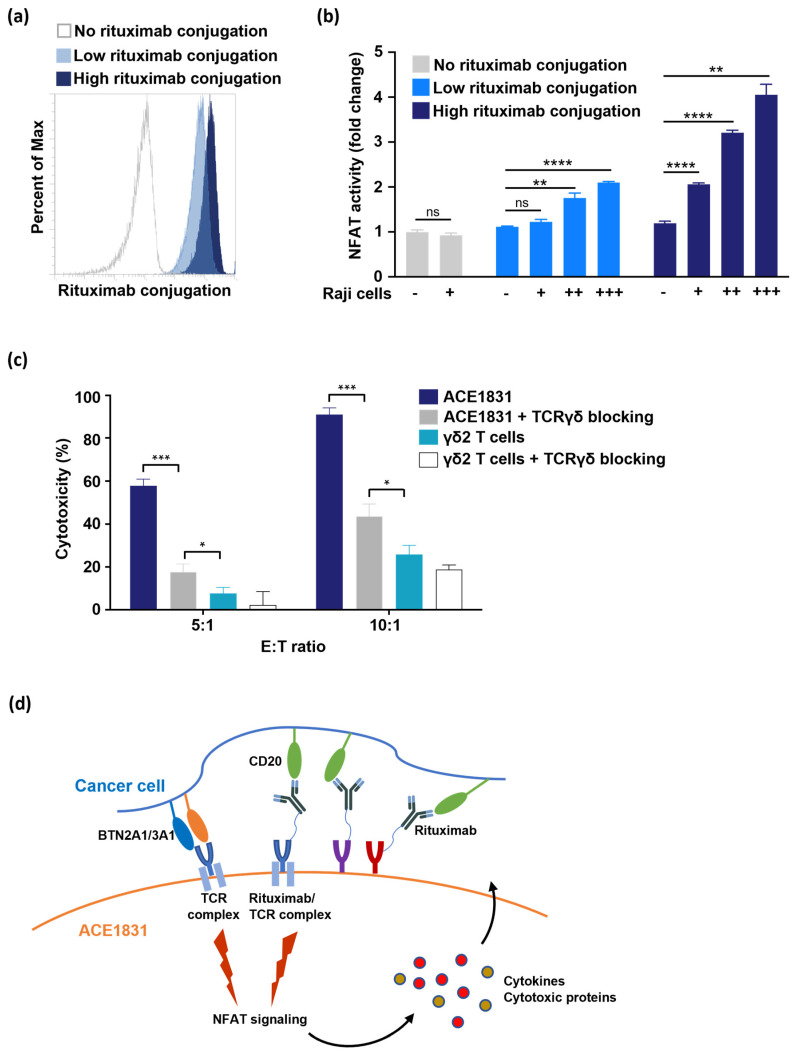
T cell activation and cytotoxicity mediated by the antigen recognition of ACC-linked antibody. (**a**) Jurkat-NFAT-Luc cells were conjugated with different amounts of rituximab using ACC technology and were stained with anti-F(ab’)2 antibody to examine the levels of rituximab conjugated on Jurkat-NFAT-Luc cells. Un-conjugated Jurkat-NFAT-Luc cells (grey line) represent negative staining, and Jurkat-NFAT-Luc cells with low (light blue) and high (dark blue) rituximab conjugation are shown. Percent of Max is the highest point of each peak of the overlaid histogram. (**b**) Jurkat-NFAT-Luc cells conjugated with different amounts of rituximab were co-incubated with different Raji cell numbers (+, 5 × 10^4^; ++, 2 × 10^5^; +++, 5 × 10^5^), and NFAT signaling activation was determined based on NFAT-regulated luciferase activity. Each condition was applied in triplicate in two different experiments, and the representative results are shown. Mean ± SD. Statistical analysis was performed using a *t* test. **, *p* < 0.01; ****, *p* < 0.0001. (**c**) The effector cells were preincubated with or without 1 μg/mL of TCRγδ blocking antibody for 1 h at 37 °C. After 4 h of co-incubation with Raji cells, cytotoxicity against Raji cells was analyzed using a CellTiter-Glo^®^ luminescent cell viability assay. Each condition was applied in triplicate in two different experiments, and the representative results are shown. Mean ± SD, *, *p* < 0.05, ***, *p* < 0.001. (**d**) Illustration delineating the activation of ACE1831 upon encountering CD20-expressing cancer cells.

The binding of receptors and ligands triggers receptor conformation change to activate downstream signaling. For example, the activation of both Vγ9Vδ2 TCR [25] and TCRαβ [26] triggers transcription factor NFAT translocation for T cell functions. However, it is not clear if the binding of ACC-linked antibodies to antigens can trigger the activation of antibody-linked receptors and downstream signaling. To examine if rituximab-linked receptors trigger downstream signaling activation when rituximab recognizes CD20, Jurkat T cells expressing a luciferase reporter driven by an NFAT-response element (Jurkat-NFAT-Luc cells) were used. Jurkat-NFAT-Luc cells were conjugated with different amounts of rituximab with ACC technology (Figure 5a) and co-incubated with Raji cells, and the luciferase activity was measured to determine if the rituximab-conjugated receptors triggered NFAT activation. The results showed that NFAT signaling was significantly activated in a dose-dependent manner when rituximab-linked Jurkat NFAT-Luc cells encountered CD20-expressing cancer cells (Figure 5b), indicating that the antigen recognition of ACC-linked antibodies activates the downstream signaling of antibody-linked receptors.

Recent findings indicated that Vγ9Vδ2 TCR senses the intracellular accumulation of phosphoantigen (pAg) bound with the BTN2A1/3A1 complex in cancer cells and activates the anti-tumor response of γδ2 T cells [27]. The mass spectrometry analysis of this study revealed that TCRδ, CD3δ and CD3γ were linked with rituximab by a DNA linker duplex. To clarify the role of rituximab-linked receptors (including TCRγδ/CD3 complex) and un-conjugated TCRγδ in ACE1831 cytotoxicity, the influence of the anti-TCRγδ blocking antibody in ACE1831 cytotoxicity was examined. The 5A6.E9 clone of the anti-TCRγδ blocking antibody has been used to block the cytotoxicity of γδ2 T cells with a limited influence on γδ T cells [28]. As shown in Figure 5c, in a pretreatment, ACE1831-mediated cytotoxicity against Raji cells was inhibited by the anti-TCRγδ blocking antibody, while it was still significantly stronger than the un-conjugated γδ2 T cells, suggesting that both the ACC-linked rituximab/receptor complex and un-conjugated TCRγδ contribute to the cytotoxicity of ACE1831 against B-cell lymphoma.

## 4. Discussion

Bio-orthogonal chemistry, based on copper-free click chemistry, has been used as a new method for the stable labeling of cells with various molecules without affecting the cell characteristics. This method has been applied for the in vivo tracking of transplanted cells in the liver and drug delivery using mesenchymal stem cells in cancer therapy [29,30]. ACC technology, developed by Hsiao et al. (patent WO2015168656), combines the rapid bio-conjugation reaction and the specificity and safety of the DNA duplex to provide a biorthogonal cell modification method. The advantage of ACC technology is its compatibility with all types of cells, which supports its potential broad applications in cell therapies, including cancer-targeting cell therapy and cell transplantation. Here, we demonstrated the compatibility of ACC technology with primary γδ2 T cells and successfully generated ACE1831 against CD20-expressing cancer cells. In addition to a superior efficacy for CD20-expressing cancer cells in vitro, the ACE1831 treatment prolonged the survival of tumor-bearing mice without causing any clinical abnormity, supporting the prospective clinical applications of ACC technology in cancer therapies.

Adoptive cell therapy using CAR-αβ T cells to treat hematologic malignancies has fundamentally transformed the treatment strategies. Nevertheless, the persistent CAR αβ T cells may have potential on-target off-tumor concern against non-malignant cells and contribute to prolonged cytopenia [31]. In addition to well-developed CAR-αβ T cells, CAR technology has recently been applied to γδ T cells [32]. A retrovirus-based second generation of the GD2- or CD19-targeting CAR construct was transduced into expanded γδ2 T cells [13,33]. Of note, the viral transduction strategy in γδ T cells is not efficient [33], even when compared to that of αβ T cells [13]. As for non-viral strategies, messenger RNA-based EpCAM-targeting CAR constructs have been delivered into Vγ9Vδ2 T cells through electroporation with a good delivery efficiency [34]. Both retrovirus- and electroporation-based CAR construct delivery systems provide γδ2 T cells with a good anti-tumor activity, whereas these genetic modification methods increase the manufacturing burden and potential safety concerns. Without genetic engineering, ACC technology was used to conjugate γδ2 T cells with a >99% rituximab conjugation efficiency to provide CD20-specific binding capacity. The augmented cytotoxicity of ACE1831 against CD20-expressing cells, but not PBMCs from healthy donors, suggests that non-genetically engineered ACC technology provides an economic and efficient method for arming γδ2 T cells with specific cancer-targeting capacity.

γδ T cells are known to sense stress conditions, including malignant transformation. Of note, most, if not all, ligands for TCR and other co-receptors on γδ T cells appear to be involved in responses to stress conditions like cellular infection or dysregulation [35]. The independence of the histocompatibility of γδ T cells assures their safety in clinical applications [36]. Furthermore, γδ T cells usually co-express other activating natural killer cell receptors, such as NKp30, NKp44 or NKG2D, which also recognize stress-induced surface molecules that are present on malignant but absent on normal cells. The accumulation of pAg, such as isopentenyl pyrophosphate, a mevalonate pathway intermediate that is up-regulated due to metabolic dysregulation in various types of tumor cells, is sensed by Vγ9Vδ2 TCR, which is only expressed by γδ2 T cells [37,38]. In contrast, normal cells do not accumulate pAg to a level sufficient to activate γδ2 T cells, which serves as at least one of the important mechanisms used to distinguish between cancer and normal cells and contributes to potential efficacy against heterogenous populations of tumor cells [39]. In this study, both ACE1831 and un-conjugated γδ2 T cells exhibited anti-cancer cytotoxicity against CD20-negative K562 cells, which provides the potential to overcome the heterogeneity or cancer marker loss of cancer cells. Additionally, we did not observe a significant toxicity of ACE1831 and γδ2 T cells to PBMCs, indicating the specificity of γδ2 T cells to transformed cells. 

Clinical trials based on adoptively transferred γδ T cells have been conducted for hematological cancers and solid tumors including lung cancer, breast cancer and hepatocellular carcinoma and demonstrated the safety and feasibility of γδ T cells for cancer treatments [5,10]. Despite the good safety profile and allogeneic application potential of γδ T cells, the limited efficacy of unmodified γδ T cell-based immunotherapy, with an average response rate of 21% during the initial clinical trials, underscores the need for further improvement [6]. In this study, we applied ACC technology to confer cancer-targeting capacity on γδ2 T cells. Rituximab was conjugated on γδ2 T cells with ACC technology, and a CD20 binding specificity, along with a strong in vitro and in vivo potency, was demonstrated. The safety of ACC linkers has been indicated in the ongoing clinical trial investigating ACE1702, a NK-based cell therapy product conjugated with the anti-HER2 antibody with ACC technology, which has no dose-limiting toxicity at doses of three billion cells/cycle [40]. These findings further support the clinical application of ACE1831 in the phase I clinical trial (NCT05653271).

Previous studies have shown that ACC technology links cancer-targeting antibodies on several types of immune cells without interfering in their intrinsic biochemistry and provides immune cells with specificity and efficacy against cancer cells [19,20]. The potential mechanism of action through which ACC-linked antibodies activate antibody-linked receptors and downstream signaling and the role of ACC-linked receptors in cytotoxicity mediated by γδ2 T cells are worth investigating. This study, for the very first time, illustrates that the ACC-linked antibody/receptor complex activates the downstream signaling of receptors upon antigen binding. One hypothesis is that the binding of the antibody to an antigen induces the conformation change in antibody-linked receptors through a DNA linker duplex or triggers receptor clustering to form an immunological synapse. Furthermore, through mass spectrometry analysis, several components of the TCR complex TCRδ, CD3δ and CD3γ were identified to be linked with antibodies through ACC technology, indicating that a portion of the TCRγδ complex was antibody-linked and could be activated by antibody–antigen binding. Based on these results and the finding that the blocking of TCRγδ signaling partially decreased ACE1831-mediated cytotoxicity, we hypothesize that the ACC-linked antibody/receptor complex is capable of recognizing cancer cells and activating ACE1831 in a prompt fashion, while the un-conjugated Vγ9Vδ2 TCR still makes a significant contribution to ACE1831 potency (Figure 5d).

## 5. Conclusions

In summary, we successfully applied ACC technology to develop a novel CD20-targeting γδ2 T cell product, ACE1831, and proved the superior in vitro and in vivo potency of ACE1831 against CD20-expressing cancer cells. In addition, the role of the antibody/receptor complex linked using ACC technology in T cell activation and potency was demonstrated. As an allogeneic γδ2 T cell therapy without genetic engineering, ACE1831 has the potential to benefit patients with relapsed/refractory B-cell lymphoma through its superior potency and off-the-shelf potential.

## Data Availability

Not applicable.

## Supplementary Materials

The following supporting information can be downloaded at: https://www.mdpi.com/article/10.3390/cancers15194844/s1, Figure S1: Resistance of rituximab-resistant Raji cells. Parental and rituximab-resistant Raji cells were co-incubated with rituximab at 0.1, 1, 10 and 100 μg/mL for 48 h. The cytotoxicity was analyzed using a CellTiter-Glo^®^ luminescent cell viability assay. Each group was tested in triplicate in two independent experiments, and the representative results are shown. Statistical analysis was performed using a *t* test. **, *p* < 0.01; ***, *p* < 0.001. Figure S2: No significant induction of cytokine release in PBMCs from healthy donors by ACE1831. ACE1831 was co-incubated with PBMCs from healthy donors (n = 4) at a ratio of 1:1 in the absence (unstimulated) and presence (stimulated) of anti-CD3/anti-CD28 beads (Gibco) for 2 or 6 days. The supernatant in each condition was collected, and the levels of IL-2 (Day 2), IFNγ, TNFα, GM-CSF, IL-4, IL-6, IL-8 and IL-10 (Day 6) were analyzed using the Human Cytokine Magnetic Bead Panel Kit according to the manufacturer’s instructions. ns, not statistically significant based on *t* test. Figure S3: In vivo potency of a single dose of ACE1831 against CD20-expressing cancer cells. Tumor-bearing SCID–Beige mice were treated with a single dose of ACE1831 (1 × 10^7^), γδ2 T cells (1 × 10^7^) and Vehicle (serum-free RPMI-1640). (A) Tumor burden of mice (n = 5 per group) was determined through bioluminescence imaging. (B) The bioluminescence intensity of the tumor burden is presented as mean values ± SD. The difference in mean tumor burden between groups was examined using two-way ANOVA. ***, *p* < 0.001. (C) The survival rate of mice with different treatments was analyzed with the Kaplan–Meier method. ACE1831 vs. Vehicle and ACE1831 vs. γδ2 T cells showed significance. **, *p* < 0.01. Figure S4. No significant toxicology of ACE1831 in SCID–Beige mice. ACE1831 (1 × 10^7^) was intravenously delivered into SCID–Beige mice on Day 0. On Day 1 (acute toxicity study) and Day 14 (recovery study), the mice (n = 5 per group per time point) were sacrificed to collect major organs (lung, heart, liver, spleen, and kidney) for organ weight measurements and blood for complete blood count analysis, respectively. Necropsy was also performed to exclude any abnormalities.
