# Supplementary material for: A Novel Allogeneic Rituximab-Conjugated Gamma Delta T Cell Therapy for the Treatment of Relapsed/Refractory B-Cell Lymphoma
Source: Cancers (Basel). 2023 Oct 4;15(19):4844. doi: 10.3390/cancers15194844 (PMC10571679; doi:10.3390/cancers15194844)
Supplement: Supplementary file 1 [file cancers-15-04844-s001.zip › cancers-2566293-supplementary.pdf]

Supplementary Figures and legends

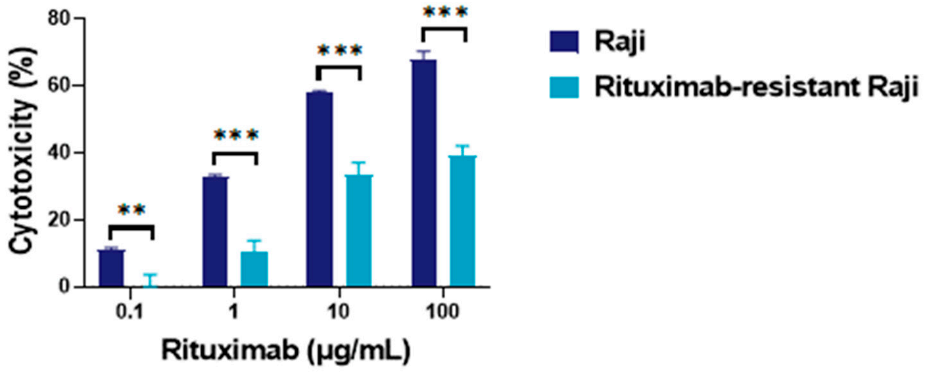

Figure S1. Resistance of rituximab-resistant Raji cells to rituximab. Parental and Rituximab-resistant Raji cells were co-incubated with rituximab at 0.1, 1, 10 and 100 μg/mL for 48 hours. The cytotoxicity was analyzed by CellTiter-Glo® luminescent cell viability assay. Each group was performed in triplicate from two independent experiments and the representative result was shown. Statistical analysis was performed by t test. **, $p < 0.01$; ***, $p < 0.001$.

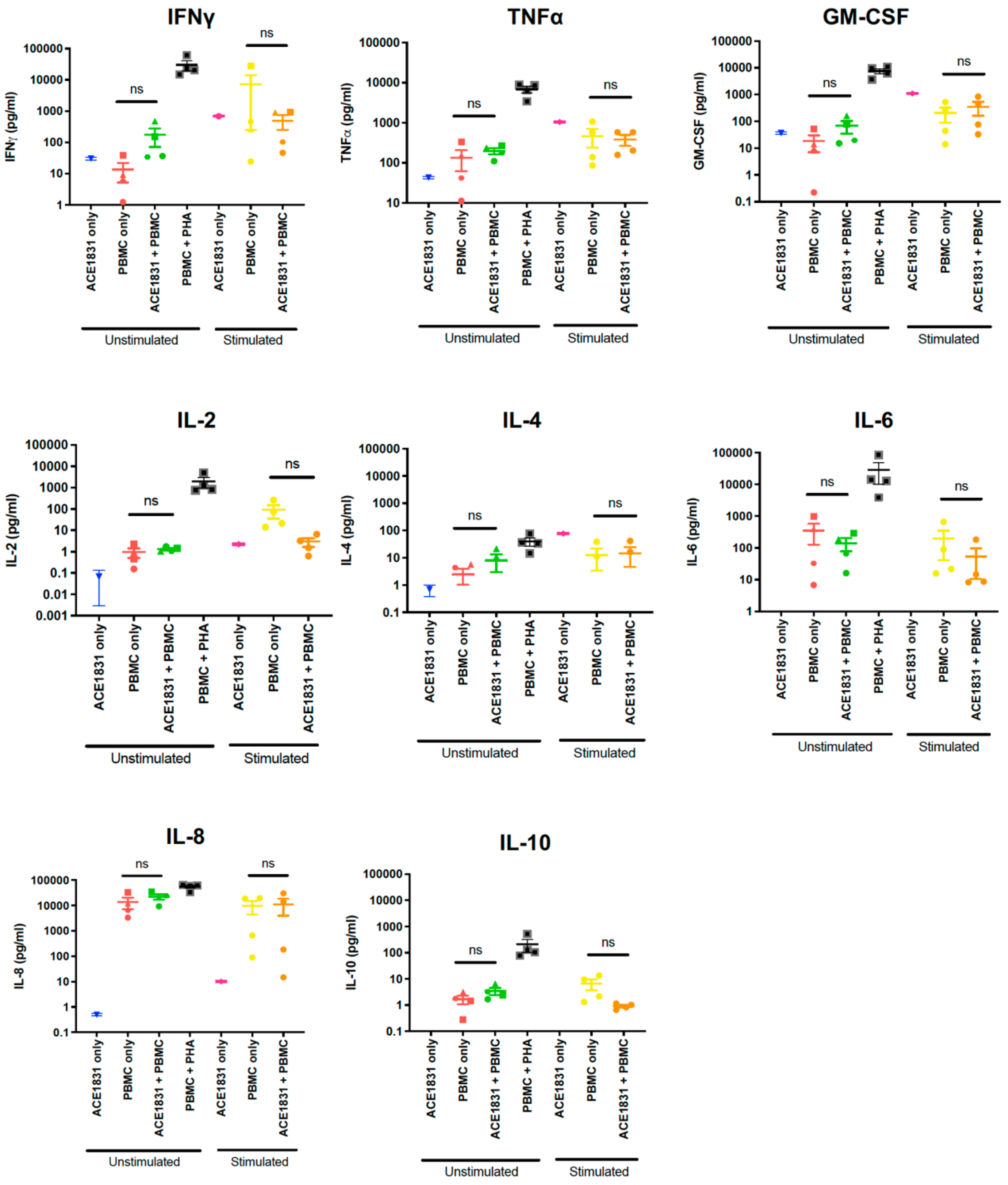

Figure S2. No significant induction of cytokine release in co-culture of ACE1831 and PBMC from healthy donors. ACE1831 was co-incubated with PBMC from healthy donors (n = 4) at ratio of 1:1 in the absence (unstimulated) and presence (stimulated) of anti-CD3/anti-CD28 beads (Gibco) for 2 or 6 days. The supernatant from each condition was collected, and the levels of IL-2 (Day 2) and IFNγ, TNFα, GM-CSF, IL-4, IL-6, IL-8, and IL-10 (Day 6) were analyzed by the Human Cytokine Magnetic Bead

Panel Kit according to the manufacturer's instruction. ns, not statistically significant by t test.

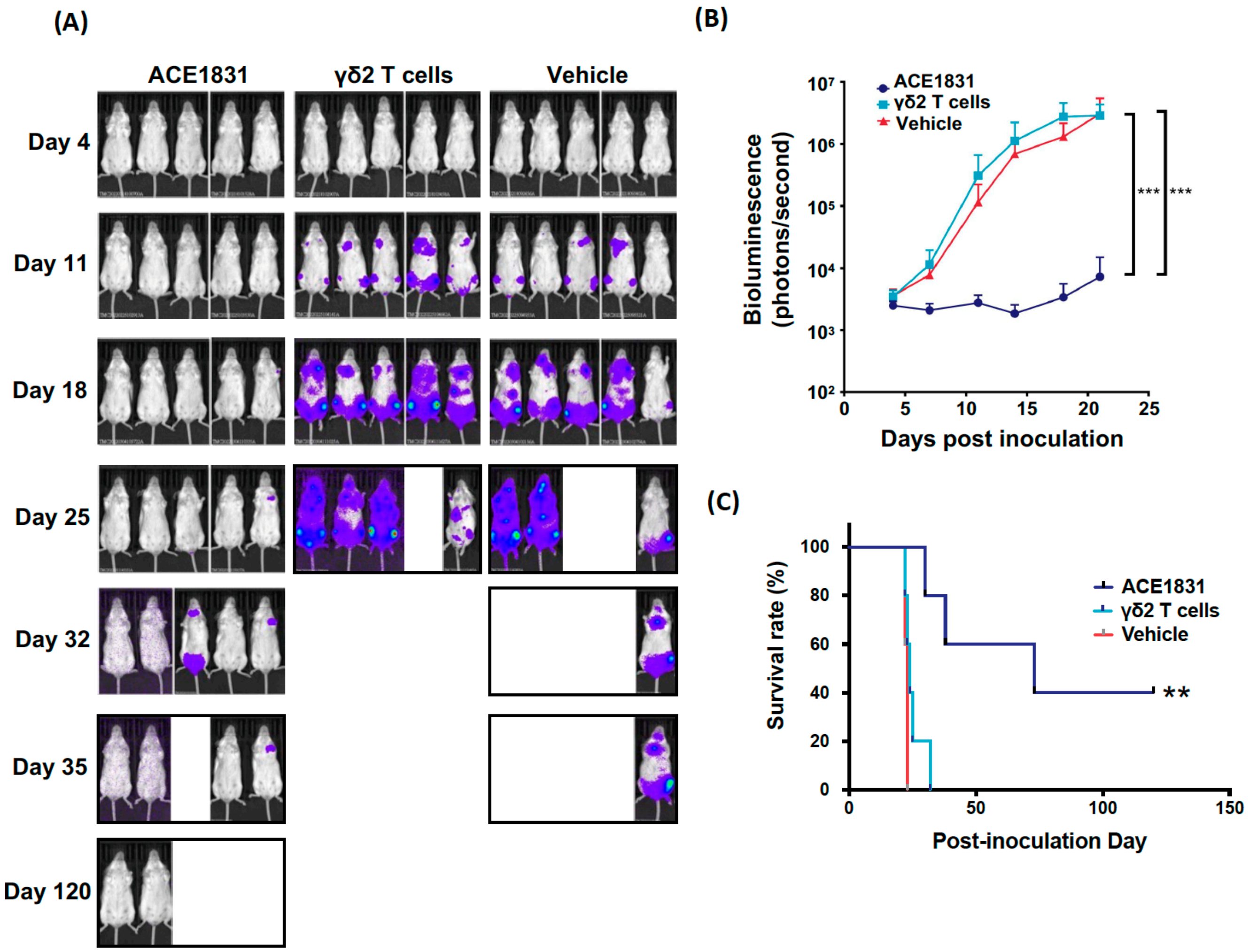

Figure S3. ***In vivo*** **potency of single dose of ACE1831 against CD20-expressing cancer cells.** Tumor-bearing SCID-Beige mice were treated with single dose of ACE1831 (1×10^7), γδ2 T cells (1×10^7), and Vehicle (serum-free medium). (A) Tumor burden of mice (n = 5 per group) was determined by bioluminescence imaging. (B) The bioluminescence intensity of tumor burden was presented by mean values ± SD. The difference in mean tumor burden between groups was examined by two-way ANOVA test. ***, $p < 0.001$. (C) The survival rate of mice with different treatments was analyzed by Kaplan-Meier method. **, $p < 0.01$.

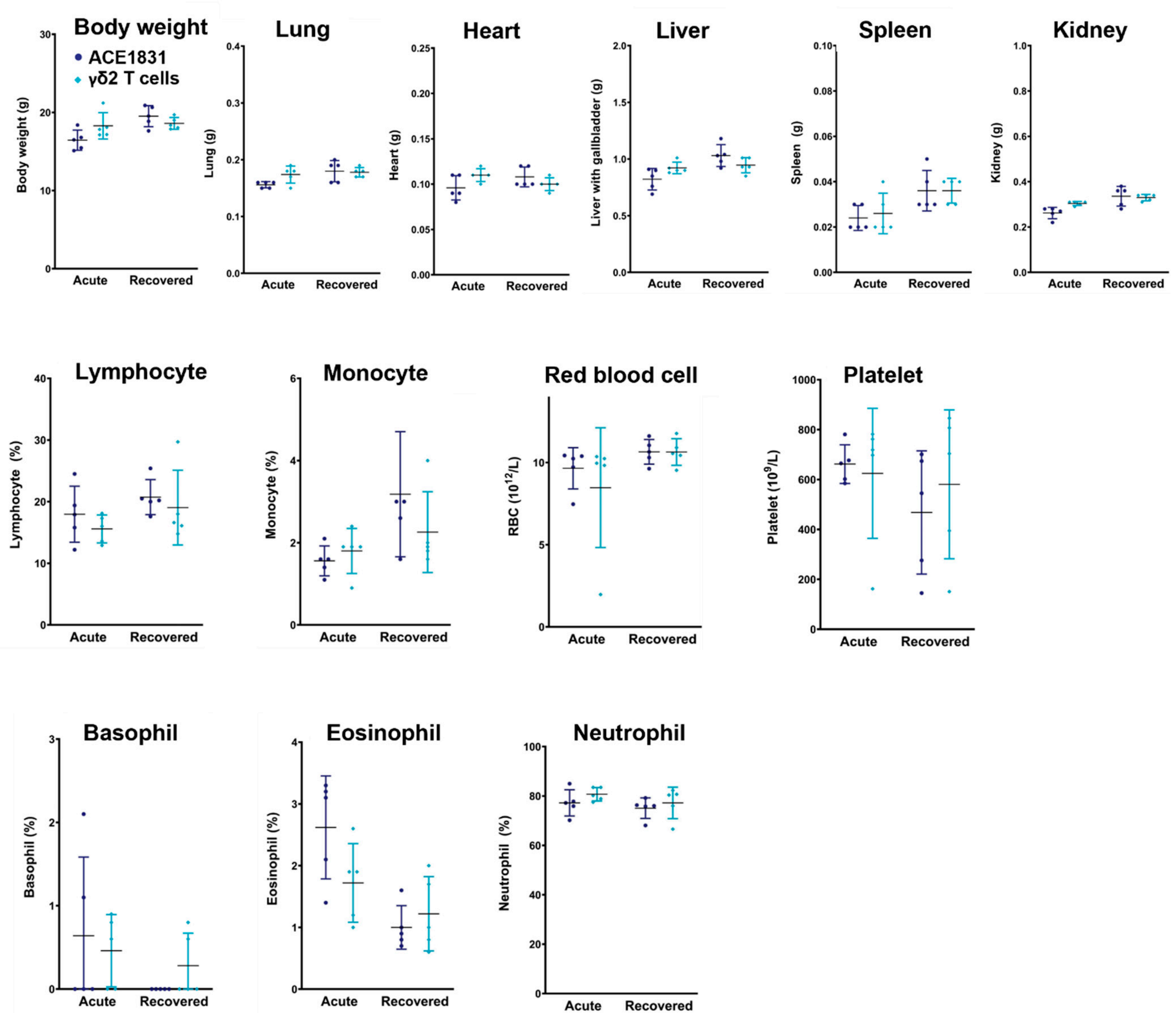

Figure S4. No significant toxicology of ACE1831 in SCID-Beige mice. ACE1831 (1×10^7) was intravenously delivered into SCID-Beige mice on Day 0. On Day 1 (acute toxicity study) and Day 14 (recovery study), mice (n = 5 per group per time point) were sacrificed to collect major organs (lung, heart, liver, spleen, and kidney) for organ weight measurement and blood for complete blood count analysis, respectively. Necropsy was also performed to exclude any abnormalities.
